# Antibody-Conjugated Paramagnetic Nanobeads: Kinetics of Bead-Cell Binding

**DOI:** 10.3390/ijms15058821

**Published:** 2014-05-19

**Authors:** Shahid Waseem, Michael A. Allen, Stefan Schreier, Rachanee Udomsangpetch, Sebastian C. Bhakdi

**Affiliations:** 1 Department of Pathobiology, Faculty of Science, Mahidol University, Rama 6 Road, Bangkok 10400, Thailand; E-Mails: swaseem92@yahoo.com (S.W.); rachanee.udo@mahidol.ac.th (R.U.); 2 Department of Physics, Faculty of Science, Mahidol University, Rama 6 Road, Bangkok 10400, Thailand; E-Mail: maa5652@gmail.com; 3 Institute for Innovative Learning, Mahidol University, 999 Phuttamonthon 4 Road, Salaya, Nakorn Pathom 73170, Thailand; E-Mail: stefan-schreier@web.de; 4 X-Zell Biotec Co., Ltd., Na-Nakorn Building, 99/349, Moo 2, Chaengwattana Road, Laksi, Bangkok 10210, Thailand

**Keywords:** high gradient magnetic separation, bead-cell binding, iron content, paramagnetic nanoparticles, magnetic labeling, cell separation

## Abstract

Specific labelling of target cell surfaces using antibody-conjugated paramagnetic nanobeads is essential for efficient magnetic cell separation. However, studies examining parameters determining the kinetics of bead-cell binding are scarce. The present study determines the binding rates for specific and unspecific binding of 150 nm paramagnetic nanobeads to highly purified target and non-target cells. Beads bound to cells were enumerated spectrophotometrically. Results show that the initial bead-cell binding rate and saturation levels depend on initial bead concentration and fit curves of the form *A*(1 − exp(−*kt*)). Unspecific binding within conventional experimental time-spans (up to 60 min) was not detectable photometrically. For CD3-positive cells, the probability of specific binding was found to be around 80 times larger than that of unspecific binding.

## 1. Introduction

Magnetic labelling of cells with paramagnetic nanoparticles is widely used in biomedical sciences. Labelled cells are used in research as well as in clinical diagnostics and therapy, facilitating a range of applications from magnetic cell separation [1] and clinical imaging to targeted drug delivery [2]. The present paper focuses on labelling of cells for magnetic separation of target cells from heterogeneous cell suspensions. For magnetic cell separation in biomedical sciences, high gradient magnetic separation (HGMS) is the most widely used technique, with over 12,000 studies published [3]. Other separation technologies include magnetohydrostatic separation [1,4–7], magnetohydrodynamic separation, and separation using eddy currents [8,9].

In most magnetic cell separation protocols, target cells are labelled with magnetic nanobeads that are conjugated to specific antibodies [10]. The efficiency of magnetic separation is influenced by factors related to the hardware of the magnetic separation system used and factors determined by the quality of the magnetic labelling of cells which depend on the properties or quality of the magnetic nanobeads employed [11]. An ideal magnetic nanobead-based cell labelling system would offer completely specific binding of beads to target cells while completely avoiding binding of beads to non-target cells [12]. To optimize a nanobead-based magnetic separation system, it is therefore crucial to understand the kinetics of specific and unspecific binding of beads to cells.

Previous studies have shown the negative impact of unspecific binding on the downstream recovery rate and purity of the target cells. It was established that optimization of bead concentration can minimize capture of non-target cells during HGMS [13,14].

The present study determines the binding rates for specific and unspecific binding of 150 nm paramagnetic beads to target and non-target cells using highly purified populations of untouched CD3- and CD14-positive cells. These cells often need to be separated in biomedical research. Hence the model is deemed relevant to a large number of biomedical studies. The model allows us to calculate the average number of beads per cell by optimized spectrophotometric measurement of total iron in well-defined populations of labelled target and non-target cells or, in other words, in models of specific and unspecific bead-cell binding.

## 2. Results

### 2.1. Characterization of Paramagnetic Nanobeads

**Figure 1.**
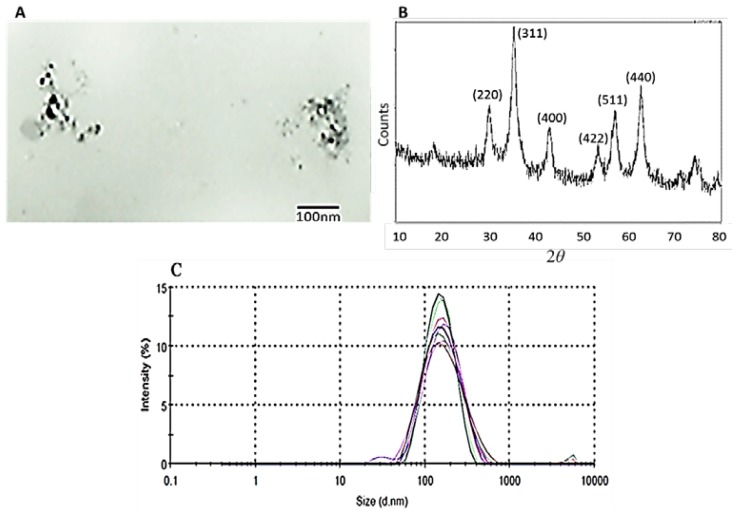
Characterization of paramagnetic nanobeads. (**A**) TEM micrograph of antibody-conjugated magnetic nanobeads. The multi-domain iron oxide cores from two nanobeads are visible. Bar size is 100 nm; (**B**) XRD pattern of antibody-conjugated magnetic nanobeads; (**C**) Size distribution of antibody-conjugated magnetic nanobeads is shown by a dynamic light scattering (DLS) graph. Ten replicates, as shown by peaks of different colours, were analysed.

Manufacturer information described beads as polymer embedded, multi-domain iron oxide cores covalently conjugated to antibodies. Transmission electron microscope (TEM) images showed the iron oxide cores of nanobeads. As expected, the polymer matrix of the nanobeads could not be visualized by TEM. From TEM imaging, the iron oxide cores are estimated to measure 30–50 nm (Figure 1A). X-ray diffraction (XRD) confirmed that the iron oxide crystals consist of pure magnetite (Fe_3_O_4_) with a characteristic pattern of peaks at angles (2*θ*) of 30.1, 35.5, 43.1, 53.4, 57.0 and 62.6 which correspond to the Miller indices shown in Figure 1B. Dynamic light scattering (DLS) of antibody-conjugated magnetic nanobeads returned a mean diameter of 158 and 156 nm for anti-CD3 and anti-CD14 beads, respectively, which is consistent with polymer embedding of magnetite crystals as seen in TEM and XRD (Figure 1C). TEM and XRD data are consistent with superparamagnetic iron oxide (magnetite) nanoparticles as described previously [15,16] and with data provided by the manufacturer. Determination of the antibody concentration of the nanobeads via a UV-Vis spectrophotometer showed 40 μg of antibodies per mg of nanobeads.

### 2.2. Enrichment of Untouched CD3- and CD14-Positive Cells by Flow Cytometry

**Figure 2.**
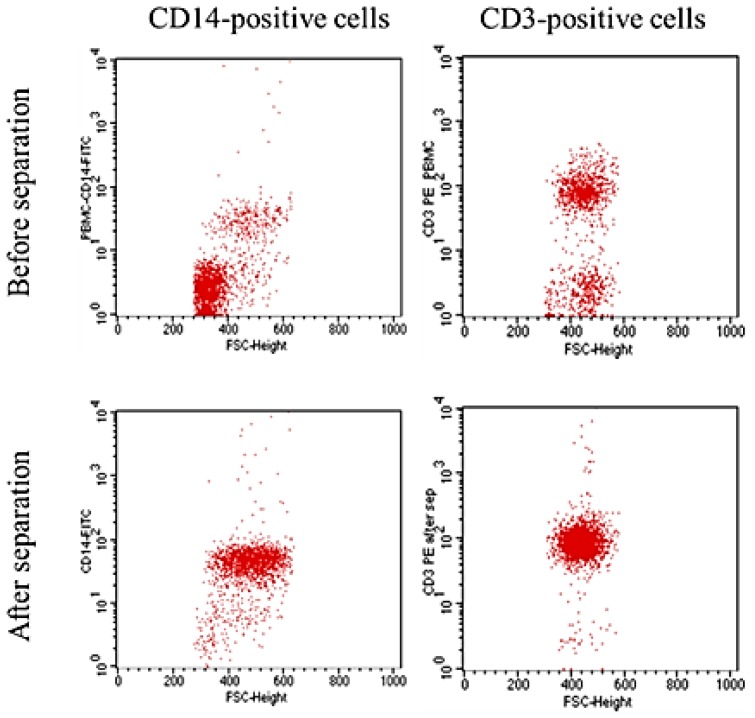
Flow cytometric analysis of untouched CD14- and CD3-positive cells separated by buffer optimized high gradient magnetic separation (HGMS) (negative selection). Plots show CD14- and CD3-positive cells before and after magnetic separation. CD14-positive cells were enriched from 17% to 95% and CD3-positive cells from 62% to 96%.

Negative isolation of untouched CD3- or CD14-positive cells was performed as described in the Experimental Section. Over 95% purity was achieved for untouched CD3- or CD14-positive cells in all the experiments. One representative experiment is shown in Figure 2.

### 2.3. Standard Curve

**Figure 3.**
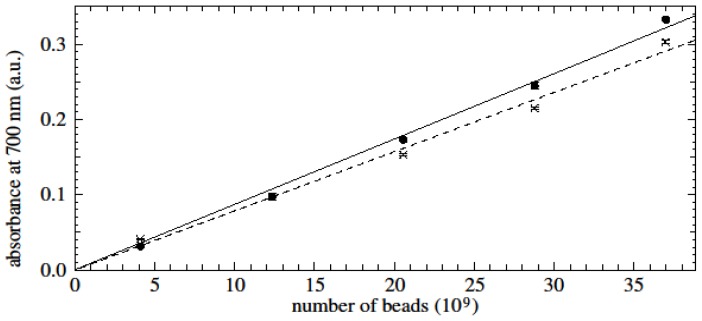
Standard curves for the number of magnetic beads with (solid line, dots) or without peripheral blood mononuclear cells (PBMC) (dashed line, crosses). For both standard curves *R*^2^ = 0.99. In this and later figures, data points are the mean of triplicate values. Vertical lines (sometimes smaller than the plotted points) indicate the range of values.

Standard curves were generated for varying amounts of magnetic nanobeads with or without peripheral blood mononuclear cells (PBMC) using the method described in the Experimental Section. Two million PBMC were used for each data point. A linear relationship was determined between absorbance and number of magnetic beads (Figure 3). The presence of PBMC did not show any significant interference with the linearity of the relationship, especially in the range that proved to be relevant for kinetic experiments (up to 10^10^ beads). Calculations for the number of beads per cell for specific or unspecific binding in the proceeding experiments were made according to the standard curve of iron with PBMC (solid line, Figure 3).

### 2.4. Quantification of Time-Dependent Binding of Magnetic Nanobeads to Target Cells

Specific binding assays were performed using anti-CD3 and anti-CD14 conjugated magnetic nanobeads with purified, untouched CD3-positive cells and CD14-positive cells, respectively. To determine the rate of unspecific binding of antibody-coated beads to cells, anti-CD3 labelled beads were incubated with untouched CD14-positive cells and *vice versa*.

Specific and unspecific binding kinetics of nanobeads to target cells were determined at time points varying from 5–45 and 30–300 min, respectively.

#### 2.4.1. Kinetics of Nanobead Binding to Untouched CD3-Positive Cells

**Figure 4.**
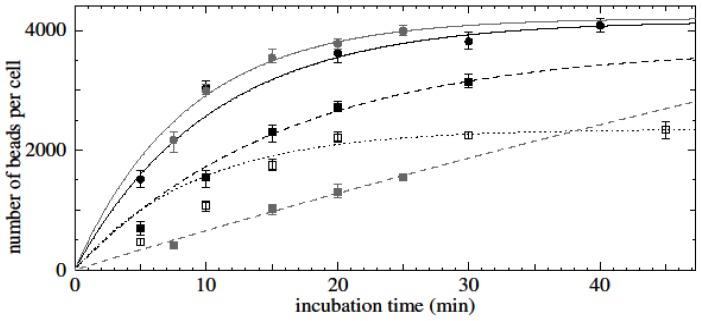
Time-dependent binding of anti-CD3 conjugated magnetic nanobeads to untouched CD3-positive cells. In this figure and the next, data are fitted to saturation-type curves of the form *A*(1 − exp(−*kt*)) where *t* is the incubation time. Solid black and grey line and round dots: 32 μg beads/100 μL, 10^6^ cells/100 μL, *R*^2^ = 0.95 and 0.99 (cells from two different donors); black dashed line and square dots: 32 μg beads/100 μL, 2 × 10^6^ cells/100 μL, *R*^2^ = 0.99; dotted line and squares: 16 μg beads/100 μL, 10^6^ cells/100 μL, *R*^2^ = 0.95; grey dashed line and square dots: 8 μg beads/100 μL, 5 × 10^5^ cells/100 μL, *R*^2^ = 0.98.

The kinetics of specific time-dependent binding of nanobeads to purified untouched CD3-positive cells was determined for different concentrations of nanobeads and target cells. The experiment with the lowest nanobead concentration (8 μg/100 μL), where the target cell concentration was maintained at 5 × 10^5^ cells/100 μL, did not reach saturation (grey dashed line and square dots, Figure 4). Nanobead concentrations of 16 μg/100 μL (dotted line, Figure 4) and 32 μg/100 μL (black dashed line, Figure 4) where target cell concentrations were maintained at 1 × 10^6^/100 and 2 × 10^6^/100 μL, respectively, reached saturation after 20 or 30 min of incubation. Interestingly, higher saturation levels were achieved for higher concentrations of nanobeads and higher bead-to-cell ratios.

The effect of different blood donors on the kinetics of bead-cell binding was assessed by keeping the concentrations of nanobeads (32 μg/100 μL) and untouched CD3-positive cells (1 × 10^6^/100 μL) constant. Similar kinetics of bead-cell binding and equal numbers of nanobeads per cell at saturation (~4000 beads per cell) were obtained for the two experiments based on blood samples from two different donors (solid lines, Figure 4).

#### 2.4.2. Kinetics of Nanobead Binding to Untouched CD14-Positive Cells

**Figure 5.**
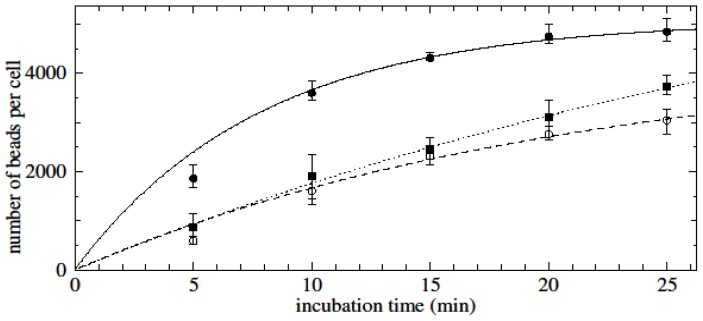
Time-dependent binding of anti-CD14 conjugated magnetic nanobeads to untouched CD14-positive cells. Solid line: 32 μg beads/100 μL, *R*^2^ = 0.98; dashed line and circles, dotted line and square dots: 16 μg beads/100 μL, *R*^2^ = 0.98 and 0.99. Concentration of target cells: 1 × 10^6^/100 μL. The experiments are from samples from three different blood donors.

Kinetics of specific time-dependent binding of nanobeads to highly pure untouched CD14-positive cells were determined for different concentrations of nanobeads keeping the target cell concentrations constant. Saturation levels of around 3500 and 5000 nanobeads per cell were obtained in experiments where the concentration of nanobeads was 16 μg/100 μL (dashed and grey lines) and 32 μg/100 μL (solid line), respectively. As with the CD3 cells, the saturation levels were higher for the larger bead-to-cell ratio case (Figure 5).

#### 2.4.3. Maximum Bead Saturation of Cells: Specific Binding Assays

**Figure 6.**
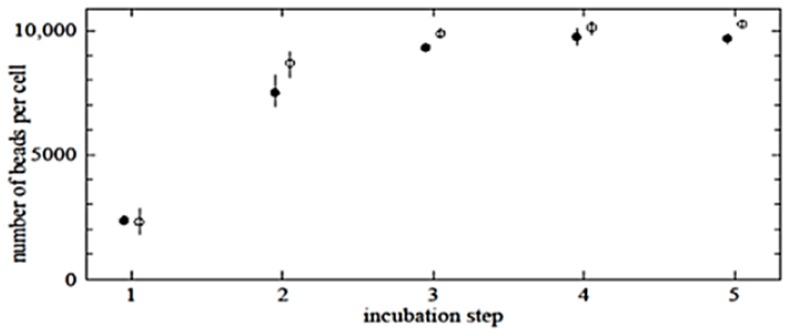
Maximum saturation level for target cells was determined by using multi-step incubation, as described in the Experimental Section. Solid and open circles represent the number of beads per untouched CD14- and CD3-positive cell, respectively.

To determine the saturation level of specific nanobead-cell binding, saturation experiments were performed by adding an excess of beads, as described in the Experimental Section. It was observed that repeated incubation (“multi-step incubation”) of cells with beads increased the number of beads per cell to approximately 10,000. After three steps of bead-cell incubation, the numbers of beads per cell did not increase further. Similar results were found for both anti-CD3 (open circles) and anti-CD14 (solid circles) conjugated magnetic nanobeads (Figure 6).

#### 2.4.4. Unspecific Binding Assays

**Figure 7.**
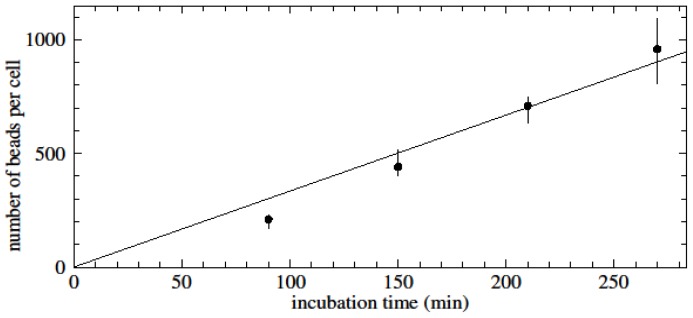
Time-dependent unspecific binding of anti-CD14 conjugated magnetic nanobeads to untouched CD3-positive cells. Concentration of nanobeads: 16 μg/100 μL; concentration of target cells: 10^6^/100 μL.

To examine the rates of unspecific binding of beads to cells, highly purified, untouched populations of CD3 positive cells were incubated with CD14-specific beads and *vice versa*. Around 1000 nanobeads per cell were obtained after 5 h of incubation (Figure 7). The binding rate for unspecific binding was found to be 3.3 beads/cell/min (*R*^2^ = 0.95). This scales to 5.0 beads/cell/min for a bead concentration of 10^12^/mL.

### 2.5. Initial Binding Rate

**Figure 8.**
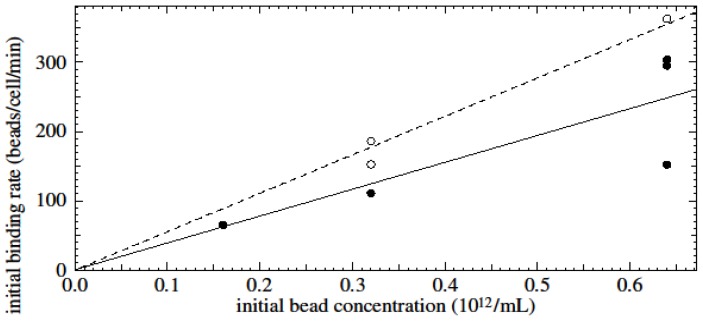
Initial rate of bead-cell binding as a function of initial bead concentration. Solid line and dots: CD3; dashed line and circles: CD14.

The initial binding rate was determined by calculating the initial slopes of time-dependent binding curves of untouched CD3- and CD14-positive cells. The initial binding rate (the number of beads binding to a cell per minute) was found to vary linearly with the initial bead concentration (Figure 8). The initial binding rate of beads to untouched CD3-positive cells (solid line, slope 390) is less than that of beads to untouched CD14-positive cells (dashed line, slope 550). Correlation of initial binding rate with initial bead concentration for CD3- and CD14-positive cells was found to be significant with *R*^2^ values of 0.69 and 0.97, respectively. The slope for specific (plus unspecific) binding to CD3 positive cells is 390/5.0 = 80 times larger than the slope would be for unspecific binding to CD3 positive cells. When there is a small amount of unspecific binding, this is roughly the ratio of probabilities for specific and unspecific binding, as is described further in the discussion.

## 3. Discussion

HGMS is the most widely used magnetic cell separation technique for the isolation of magnetically labeled cells from cell suspensions [17]. A cell labelled with magnetic nanobeads will be captured in the HGMS column if the magnetic force acting on the cell is larger than the drag and gravitational forces [8]. In principle, the magnetic force acting on the cell is proportional to its magnetic susceptibility [18], and the magnetic susceptibility of a cell is proportional to the number of magnetic nanobeads bound to the cell.

To optimize any magnetic separation assay it is therefore crucial to understand the kinetics of specific and unspecific bead-cell binding in detail. Previously, unspecific bead-cell binding was examined as a function of bead concentration for different cell types [14]. However, to our knowledge, this is the first time that bead-cell binding kinetics has been examined by direct quantification of magnetic beads on populations of highly purified target and non-target cells. For both specific and unspecific binding assays, sensitive iron detection assays were adopted to determine average numbers of magnetic beads per cell.

CD3-positive cells encompass the entire lymphocyte population present in PBMC, whereas CD14-positive cells are known as monocytes. Both cell types were previously described as expressing 80,000–120,000 receptors per cell [19–24]. Lymphocytes are usually 8–12 μm across, whereas monocytes exist in two well-characterized populations with diameters of 8–10 and 16–20 μm [19–24]. The ratio of the two monocyte populations can be assumed to be 2:1 (smaller:larger) in healthy individuals [19,23–25].

The antibody-conjugated magnetic nanobeads employed in the study were found to be 150–160 nm in diameter with magnetite crystal cores of 30–50 nm, as determined by DLS and TEM images.

Results showed that the initial binding rate of beads to cells is proportional to the initial bead concentration employed during bead-cell incubation. Experimental data of time-dependent assays showed saturation-type curves for all cell- and bead-type combinations.

We assume that the rate at which beads bind to cells (d*b*/d*t* where *b* is the number of beads per cell) is proportional to the concentration of viable beads in solution (which is *n*_0_ − *n*_c_*b* where *n*_0_ is the initial bead concentration and *n*_c_ is the concentration of cells) and to the remaining area on the cell that can be occupied by beads (which is proportional to *b*_max_ − *b* where *b*_max_ is the maximum possible number of beads per cell). Hence d*b*/d*t* = *C*(*n*_0_ − *n*_c_*b*)(*b*_max_ − *b*) where *C* is the constant of proportionality which is itself proportional to the probability of binding. The solutions to this equation are saturation type curves which have an initial value of d*b*/d*t* (the initial binding rate when *b* is still small) of *Cn*_0_*b*_max_. One would therefore expect that the initial binding rate is proportional to the bead concentration.

Later during the course of bead-cell binding, the value of *b* levels off at *b*_max_ when *n*_0_ > *n*_c_*b*_max_ (*i.e*., when there are enough beads in solution to saturate the cells) and at *n*_0_/*n*_c_ when *n*_0_ < *n*_c_*b*_max_ which is when there are not enough beads to saturate the cells. For the cases *n*_0_ > *n*_c_*b*_max_ and *n*_0_ < *n*_c_*b*_max_, these saturation curves are approximately of the form *A*(1 − exp(−*kt*)). Indeed, as shown in the results, fitting saturation-type curves of the form *A*(1 − exp(−*kt*)) to the experimental data returned significant *R*^2^ values, corroborating the assumption of classical, concentration-dependent saturation-type kinetics based on the theory outlined here.

To determine the maximum possible number of beads per cell, multi-step incubation experiments were performed. These gave higher saturation levels than single-step incubation experiments with increased bead concentrations (data not shown). Regarding the cells as spheres, the cell surface area is 4*πR^2^* where *R* ~5 μm is the radius of a cell, and treating the attached nanobeads conjugated to antibodies as discs of radius *r* ~80 nm, it would appear that the maximum number of beads on a cell surface would be 4*πR*^2^*f*/(*πr*^2^) where *f* is the maximum fraction of the cell surface area that can be covered by beads without overlap. Assuming that $f=\pi /\sqrt{12}=0.91$, the value for close packing of discs on a plane, gives in the order of 14,000 beads per cell. Saturation levels in multi-step incubation experiments reached approximately 10,000 beads per cell, which seems consistent with the above calculation allowing for the fact that there is a distribution of cell and bead sizes. This also corresponds with previous studies that demonstrated steric saturation of cell surfaces with nanobeads [13].

Perhaps the more interesting observation is that experiments without multi-step binding protocol show slowing of bead-binding rates and apparent saturation at much lower levels of beads per cell. A possible explanation is that the quality of beads is probably not consistent even within the same batch. Firstly, during production of nanometre-sized particles, the number of functional groups essential for antibody conjugation is difficult to control per particle, as assays of individual particles are not yet possible. Secondly, after antibody-bead conjugation, orientation and functionality of antibodies is also random to a certain extent, which further increases variability between beads. Hence it can be assumed that during the incubation of a seemingly homogenous population of nanobeads with cells, high-quality (*i.e*., highly reactive) beads are depleted first, and leftover lower quality beads contribute to a progressively slowing bead-cell binding rate, which would result in the case of *n*_0_ < *n*_c_*b*_max_, even if only looking at bead numbers would lead to the initial assumption *n*_0_ > *n*_c_*b*_max_.

This unknown distribution could partly be characterized by a combination of the change of the binding rate over time, the ratio of the maximum saturation levels in multi-step incubation experiments, and saturation levels observed in kinetic, single-incubation experiments. Elucidating further parameters to characterize the distribution in detail certainly warrants further research, since its better understanding harbours the potential to advance validation of assays involving binding of nanobeads to cells.

## 4. Experimental Section

### 4.1. Preparation of Mononuclear Cells

Whole blood was obtained from healthy donors after informed consent. PBMC were prepared by density gradient centrifugation as described previously [26]. Briefly, whole blood was mixed with an equal volume of PBS (Biochrom, Berlin, Germany). Subsequently, blood was pipetted on top of an equal volume of Lymphoprep (Axis-Shield PoC AS, Oslo, Norway) and centrifuged at 800× *g* for 20 min at 20 °C. PBMC were collected at the interface and then washed twice with PBS at 300× *g* for 10 min at 20 °C. Cell concentration was determined by haemocytometer (Boeco, Hamburg, Germany) using the trypan blue (Gibco, Life Technologies, Stockholm, Sweden) exclusion method.

### 4.2. Characterization of Paramagnetic Nanobeads

The 150 nm HMX anti-human anti-CD3, anti-CD14, and anti-biotin magnetic beads were from X-Zell Biotec, Bangkok, Thailand. According to the manufacturer, antibodies were conjugated to carboxyl-functionalized polysaccharide beads containing a multi-domain magnetite core by carbodiimide chemistry. The size distribution, morphology, and crystallinity of the nanobeads were determined by dynamic light scattering (DLS), transmission electron microscopy (TEM), and X-ray diffraction (XRD), respectively. For the DLS, the bead suspension was analysed in a Zetasizer (Malvern Instruments Ltd., Worcestershire, UK). For the TEM, an aqueous solution of the nanobeads was dispersed on a copper grid, dried under vacuum, and micrographs were recorded using a Hitachi-600 electron microscope at 80 kV. The XRD experiment was performed using a Rigaku (TTRAX III) X-ray diffractometer with fixed monochromater at a wavelength and speed of 0.1542 nm and 3°/min, respectively.

The amount of antibody on the beads was determined by a Bradford assay. Briefly, antibody-conjugated nanobeads were placed in Bradford solution for 60 min and the protein concentration was determined using a NanoDrop spectrophotometer ND-1000 (Thermo Fisher Scientific, Waltham, MA, USA) at 595 nm.

### 4.3. Isolation of Untouched CD3- and CD14-Positive Cells

#### 4.3.1. Magnetic Labeling

Untouched CD3- or CD14-positive cells were isolated from PBMC using buffer-optimized HGMS, anti-biotin magnetic beads, and a biotinylated antibody cocktail. The cocktail contained anti-CD14, -CD16, -CD19, -CD123, -CD235a for untouched CD3-positive cells, and anti-CD3, -CD7 -CD16, -CD19, -CD56 -CD123, -CD235a for untouched CD14-positive cells. All reagents were from X-Zell Biotec, Bangkok, Thailand. Briefly, PBMC were resuspended in HGMS buffer (3% BSA/PBS, pH 7.4) and incubated with human TruStain FcX (BioLegend, San Diego, CA, USA) (5 μL per 2 × 10^6^ cells) for 5–10 min at 4 °C to block Fc receptors. 10 μL of biotinylated antibody cocktail (for untouched CD3- or CD14-positive cells) was added and incubated for 10 min at 4 °C. Finally, anti-biotin magnetic beads were mixed and incubated for 15 min at 4 °C. The incubation mixture was shaken every 5 min by finger tapping and finally washed (300× *g* at 4 °C for 10 min).

The incubation volume was maintained at 250 μL. Fresh, filtered, cold buffer (3% BSA/PBS, pH 7.4) was used in the assay.

#### 4.3.2. Magnetic Isolation of Untouched CD3- and CD14-Positive Cells

Magnetically labeled PBMC were resuspended in HGMS buffer (3% BSA/PBS, pH 7.4) (500 μL/10^7^ cells) and subjected to magnetic separation as described previously [27]. Briefly, the HGMS column was filled with HGMS buffer. Air bubbles were removed by gentle finger tapping. The HGMS column was placed inside an HGMS magnet 5 min before loading the sample. The HGMS column was connected to a 26G/½-inch needle via a stopcock. Magnetically labelled PBMC were loaded onto the column while the stopcock was opened. The column was washed with 8–10 mL buffer (0.5% BSA/PBS, pH 7.4) at a flow rate of 0.33 mL/min. The target cells (untouched CD3- or CD14-positive cells) were allowed to flow through. The flow-through was centrifuged and target cells were pelleted at 300× *g* for 10 min at 4 °C. The concentration of the target cells was measured using a haemocytometer by the trypan blue exclusion method. Fresh, filtered, cold buffers (3% BSA/PBS or 0.5% BSA/PBS; pH 7.4) were used in the assay. The HGMS columns and magnet were from X-Zell Biotec, Bangkok, Thailand.

### 4.4. Flow Cytometry

The purity of the untouched CD3- or CD14-positive cells was determined using a FACScan flow cytometer (BD, Erembodegem, Belgium). Cells were labelled with anti-CD3 or anti-CD14 antibodies (Exbio, Prague, Czech Republic) conjugated with phycoerythrin (R-PE) or fluorescein (FITC) (Innova Biosciences, Cambridge, UK). Cells were analysed before and after magnetic separation to confirm enrichment. 10,000 events were acquired from each sample. Unstained untouched CD3- or CD14-positive cells (after magnetic separation) were used as a negative control.

### 4.5. Quantification of the Average Number of Beads per Cell

#### 4.5.1. Standard Curve

Non-coated 150 nm HMX beads were used in the assay (X-Zell Biotec, Bangkok, Thailand). The bead diameter was 150 nm and the bead concentration was 4.11 × 10^9^ beads/μg. The average number of beads per cell was quantified by creating standard curves of known numbers of beads using a photometric iron quantification assay as described previously [4].

Briefly, magnetic beads in distilled water containing 1, 3, 5, 7 and 9 μg beads were placed in 1.5 mL centrifuge tubes (SPL Lifesciences, Gyeonggi-Do, Korea) and PBMC were added as indicated. The bead-cell mixture was dried at 70 °C overnight. After the liquid evaporated completely, dried magnetic beads were soaked in 100 μL HCl (5M) (Thermo Fisher Scientific, Waltham, MA, USA), vortexed, and incubated at 60 °C for 4 h. Caps of the tubes were kept on to avoid evaporation. After incubation, two-fold dilution was made with freshly prepared 5% potassium hexacyanoferrate (II) (Fluka AG, Buchs, Switzerland) and the tubes were incubated for a further 35 min at room temperature in the dark. The mixture was centrifuged at 9700× *g* for 10 min. The iron content was quantified by measuring the absorption in a NanoDrop spectrophotometer ND-1000 (Thermo Fisher Scientific, Waltham, MA, USA) at 700 nm. Triplicate samples were analysed.

#### 4.5.2. Magnetic Beads per Cell

##### Kinetics of Bead-Cell Binding

To determine the number of magnetic beads on untouched CD3- or CD14-positive cells, the untouched CD3- or CD14-positive cells (2 × 10^6^ cells, and in one case, 5 × 10^5^ cells) were incubated with anti-CD3 or anti-CD14 magnetic beads (X-Zell Biotec, Bangkok, Thailand), respectively, in 5% BSA/PBS at 4 °C for 5–45 min. The incubation volume was maintained at 200 μL. Each cell suspension was agitated every 5 or 10 min by gentle tapping.

At the end of the incubation period, the cell suspension was diluted up to 10 mL and washed twice at 300× *g* for 10 min at 4 °C to wash away unbound magnetic beads. The cells were then pelleted, dried at 70 °C overnight, and the average number of beads was obtained by determining the total iron content as described in 4.5.1.

##### Maximum Bead Saturation: Specific Binding Assay

To determine the maximum saturation level per target cell, the following protocol was established. Saturation curves for specific binding for highly pure untouched CD3- or CD14-positive cells were determined by incubating target cells with anti-CD3 or anti-CD14 magnetic beads, respectively. Five sample tubes (1, 2, 3, 4 and 5) were prepared and subjected to one, two, three, four, and five incubation steps, respectively. The time for each incubation step was set at 30 min. The total amount of magnetic beads used for one, two, three, four, and five incubation steps was, respectively, 16, 64, 80, 96 and 112 μg per 1 × 10^6^ target cells per 100 μL. After the first incubation step, target cells were pelleted and resuspended in buffer, and the magnetic beads were mixed and incubated for the next 30 min at 4 °C. The incubation mixture was agitated gently every 10 min. All remaining incubation steps were performed in the same way.

##### Maximum Bead Saturation: Unspecific Binding Assay

To investigate the maximum saturation level per non-target cell (unspecific binding), anti-CD3 magnetic beads were incubated with untouched CD14-positive cells (2 × 10^6^ cells) and *vice versa*. The iron content was determined and the protocol was followed as described above.

## 5. Conclusions

The results demonstrated that the initial binding rate of magnetic nanobeads to cells is proportional to the initial bead concentration. The measured numbers of bound beads per cell as a function of time fit saturation-type curves of the form *A*(1 − exp(−*kt*)), and levels of binding after single-step incubation experiments are significantly lower than the levels achieved in multi-step incubation assays, which might be attributed to a previously unknown bead quality distribution. Our results show that the probability that a bead binds specifically is around 80 times that for unspecific binding. This explains our observation that unspecific binding within conventional experimental time-spans (up to 60 min) was not detectable photometrically.
